# Recruitment hotspots and bottlenecks mediate the distribution of corals on a Caribbean reef

**DOI:** 10.1098/rsbl.2021.0149

**Published:** 2021-07-14

**Authors:** Peter J. Edmunds

**Affiliations:** Department of Biology, California State University, 18111 Nordhoff Street, Northridge, CA 91330-8303, USA

**Keywords:** Scleractinia, coral reef, restoration, Caribbean, demography

## Abstract

Recruitment hotspots are locations where organisms are added to populations at high rates. On tropical reefs where coral abundance has declined, recruitment hotspots are important because they have the potential to promote population recovery. Around St. John, US Virgin Islands, coral recruitment at five sites revealed a hotspot that has persistent for 14 years. Recruitment created a hotspot in density of juvenile corals that was 600 m southeast of the recruitment hotspot. Neither hotspot led to increased coral cover, thus revealing the stringency of the demographic bottleneck impeding progression of recruits to adult sizes and preventing population growth. Recruitment hotspots in low-density coral populations are valuable targets for conservation and sources of corals for restoration.

## 1. Introduction

Population growth is promoted by recruitment [1,2] that can involve organisms of sexual or asexual origins [3]. The importance of each reproductive mode depends on context and taxon [4], but where recruits originate from dispersive propagules, their delivery, settlement, and post-settlement success mediate population growth [2,5]. While passive dispersal and settlement are common for many organisms [6,7], the settlement of animal propagules usually is deterministic at small spatial scales [8,9], even if large-scale dispersal reflects environmental conditions [6]. Together, these events can lead to ‘hotspots’ of biodiversity or recruitment where values for these state variables are high relative to other areas [10,11].

Recruitment hotspots have ecological relevance if, for example, they mediate population dynamics over areas larger than the hotspot [12]. They have statistical meaning if the density of recruits in the hotspot represents an upper outlying value [13]. Ecologically important recruitment hotspots have been described in multiple systems, including tropical fishes [14,15], temperate crustaceans [16] and long-lived trees [17]. Widespread community degradation underscores the importance of locations where recruitment continues to be high (i.e. hotspots).

Coral reefs are infamous for the large extent to which they have changed [18–20], and for reef taxa that have declined in abundance, recruitment hotspots have the potential to support population recovery. Fish ecologists have been identifying recruitment hotspots on reefs for decades [14,15], but coral biologists have been slower to this task, in part because coral recruitment usually is measured using settlement tiles that are challenging to deploy. Coral recruitment can be recorded photographically [21], but this approach has limited resolution [22]. Using settlement tiles over 3 years, Eagle *et al*. [23] found genus-specific settlement hotspots for corals at One Tree Reef, Great Barrier Reef (GBR). Coral recruitment hotspots have also been detected using tiles in the Red Sea [24], on the GBR [25] and in the Persian Gulf [26], and within photoquadrats over 30 years at Heron Island [21]. Other studies using tiles have shown spatial variation in coral recruitment to differ among years [27,28].

This study evaluates recruitment of corals having mostly a brooding life-history strategy. Using three decades of surveys from St. John, US Virgin Islands, I tested for hotspots in densities of coral recruits and juvenile corals (≤4 cm diameter) at scales of tens to hundreds of metres. Hotspots for recruitment and juvenile corals were detected, but their ecological impacts were truncated by processes preventing small corals from growing into adult colonies, uncoupling the two types of hotspots and preventing them from increasing coral cover. These processes create demographic bottlenecks constricting the transition of recruits to older size classes, and impeding population growth [29].

## 2. Methods

### (a) Recruitment

**Figure 1.**
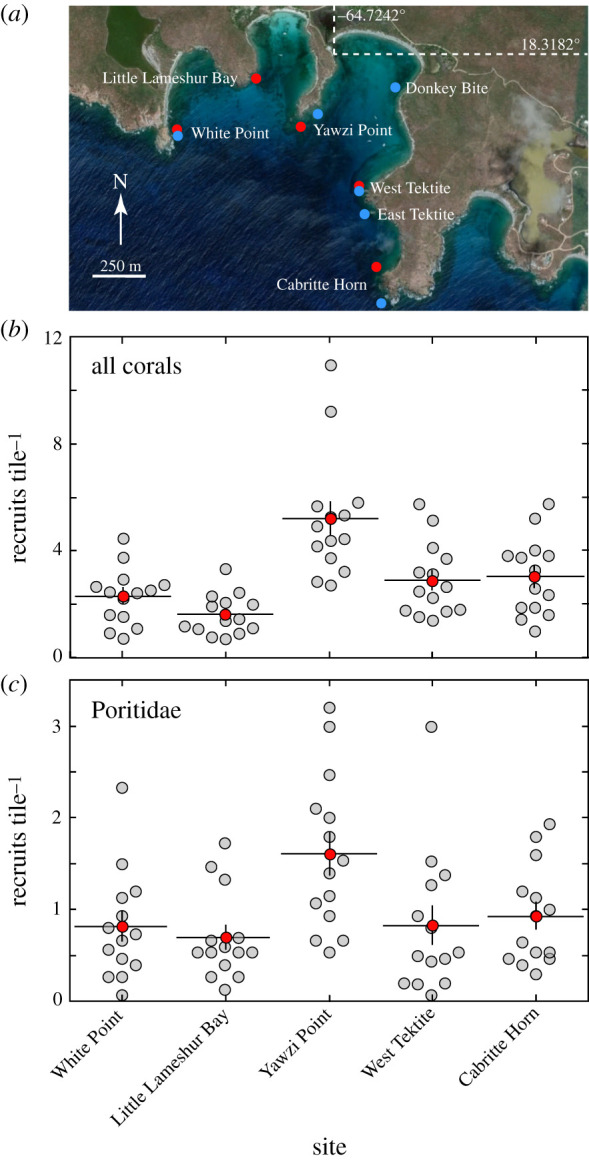
Study sites and density of recruits. (*a*) Location of sites (red = tiles, blue = juveniles), dashed lines show latitude/longitude of dock (credit: Google Earth). (*b*) Density of corals (pooled among taxa), and (*c*) density of poritids. Grey = mean recruitment by site and year; red = recruitment averaged over years, by site (±s.e., *n* = 14 years).

Coral settlement was measured using terracotta tiles (15 × 15 × 1 cm) that were individually attached to the reef at approximately 5 m depth at five sites (red points in figure 1*a*), where they were approximately 2–100 cm apart as dictated by attachment locations. The first tiles were deployed in August 2006, immersed for approximately 6 months, and replaced in 1/2007, 8/2007, 1/2008 and 8/2008; thereafter, they were immersed for approximately 12 months and replaced in July/August. Ten tiles site^−1^ were deployed per site in the first year, with 15 tiles site^−1^ in other years. Deployments in the first two years were part of a separate project [30], and in remaining years, they were coincident with annual sampling. Tiles were seasoned in seawater for 6–12 months before installation and were attached horizontally with a gap of approximately 1 cm beneath [31].

Freshly collected tiles were bleached, dried and inspected for recruits (40× magnification). Corals were identified to family (Poritidae, Faviidae, Agaricidae, Siderastreidae, Acroporidae and unidentified) and expressed as corals tile^−1^ for all corals (i.e. pooled taxa). Since tiles were immersed for approximately 6 months over the first 2 years, mean settlement by deployments was summed within year by site, which precluded calculating within-site variability. Tiles were independent in the 12 months deployments, and means (±s.e.) were calculated by year with sites as replicates (*n* = 5).

### (b) Juvenile corals

Juvenile corals were ≤4 cm diameter and were surveyed from 1994 to 2020 at six sites (blue points in figure 1*a*). Juveniles were unlikely to be sexually mature and were identified assuming maturity at approximately 4 cm diameter [32]. *Favia fragum* and *Siderastrea radians* probably achieved maturity at less than 4 cm [32] and were excluded from the analyses.

Juvenile corals were counted in quadrats (0.5 × 0.5 m, *n* = 40) randomly placed along one 40 m transect site^−1^, with surveys conducted in May (1995–1995) or July/August (1994 and 1998–2020). Corals were identified to species or genus, and the results pooled among taxa. In another study [33], small corals were tagged at each site, and their annual mortality (pooled by taxon) averaged across years by site was used to evaluate whether variation in density was associated with mortality.

### (c) Analysis

The density of recruits (pooled taxa and Poritidae) over 2007–2020 was compared among sites using Friedman's test, in which sites were replicates. The density of juvenile corals was summarized by year and site and Friedman's test was used for 1999–2020, in which all sites were sampled. Densities of juveniles were summarized by decade to test for differences using a two-factor ANOVA in which site and decade were fixed effects; assumptions were tested through analyses of residuals. To detect hotspots in densities of juveniles over metres, densities by quadrat were qualitatively compared along transects and among years at each site. Quadrats were randomly placed, but their relative positions along transects were similar among years. Annual mortality of juvenile corals was compared among sites using one-way ANOVA and logit-transformed data. Concordance in hotspots for recruits and juveniles was tested using Pearson correlations and a Mantel test comparing dissimilarity (by Bray Curtis) matrices based on mean densities for the four sites at which recruits and juveniles were measured. Statistical analyses were completed using Systat 13 and XLSTAT software.

## 3. Results

### (a) Recruitment

Recruits were found on most tiles, with a grand mean (pooled taxa) of 3.01 ± 0.22 corals tile^−1^ (range 0.73–10.93, ± s.e., *n* = 70). Poritids were the most common recruit (0.97 ± 0.09 corals tile^−1^), with densities by site within years ≥0.07 corals tile^−1^. Mean densities of five other genera ranged from 0.02 ± 0.01 corals tile^−1^ (Acroporidae) to 0.76 ± 0.09 corals tile^−1^ (Siderastreidae), and families ranked in mean abundance as Poritidae > Siderastreidae > Agaricidae > Faviidae > Acroporidae; abundance (±s.e.) of unidentifiable corals was 0.51 ± 0.07 corals tile^−1^.

Recruitment varied among sites over 2007–2020 (*F*_4,52_ = 26.014, *p* < 0.001) and was lowest at Little Lameshur Bay (mean ± s.e. = 1.64 ± 0.20 corals tile^−1^) and greatest at Yawzi Point (5.20 ± 0.62 corals tile^−1^) (figure 1*b*). Recruitment was higher at Yawzi Point than at other sites (*p* ≤ 0.001), lowest at White Point and Little Lameshur Bay (which could not be distinguished, *p* = 0.074) and intermediate at West Tektite and Cabritte Horn (which could not be distinguished, *p* = 0.813). The results for poritids (figure 1*c*) were similar, with densities differing among sites (*F*_4,52_ = 19.439, *p* = 0.001). Mean (±s.e.) poritid recruitment was lowest at Little Lameshur Bay (0.69 ± 0.13 corals tile^−1^) and greatest at Yawzi Point (1.61 ± 0.23 corals tile^−1^).

### (b) Juvenile corals

**Figure 2.**
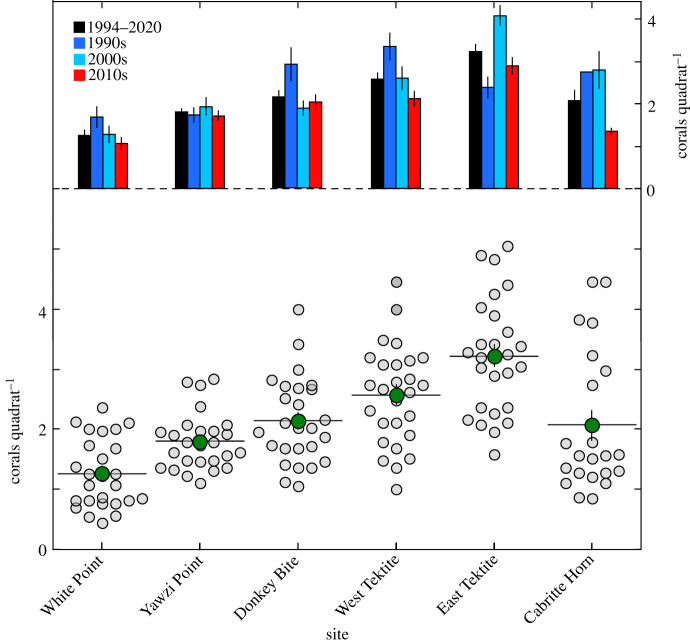
Density (corals quadrat^−1^, with a quadrat size of 0.25 m^2^) of juveniles at six sites from 1994 to 2020. Dot plots (left ordinate) show density by year and site (grey) and the mean (±s.e.) across years by site (green). Bar graphs (right ordinate) show mean (±s.e.) density by sites and decade.

The density of juveniles varied among sites (figure 2), and the most common taxon was *Porites*, which accounted for 62 ± 2% (mean ± s.e.) of corals. Mean densities (±s.e., pooled taxa) varied from 1.26 ± 0.11 colonies quadrat^−1^ at White Point to 3.22 ± 0.18 colonies quadrat^−1^ at East Tektite, and differed among sites (*F*_5,105_ = 62.295, *p* < 0.001), with relative differences that were unlike those of the recruits (figure 1). Densities of juveniles were higher at East Tektite than other sites (*p* < 0.001), and lower at White Point than other sites (*p* < 0.001). Instead of a recruitment hotspot at Yawzi Point, the hotspot for juveniles was at East Tektite, 600 m southeast (figure 2). For White Point, Yawzi Point, West Tektite and Cabritte Horn, where recruits and juveniles were both measured from 2007 to 2020, their densities were unrelated for all corals and poritids (*r* ≤ 0.078, d.f. = 54, *p* > 0.568). Similarities among sites by mean densities of recruits and juveniles were unrelated for all corals (*r*(AB) = −0.190, *p* = 0.613) and for poritids (*r*(AB) = −0.488, *p* = 0.265).

The among-site differences in density of juveniles remained evident by decade, but densities declined over time. Relative to the decade with the highest density (1990s or 2000s), mean density in the 2010s was depressed by 11% (Yawzi Point) to 52% (Cabritte Horn). Density was affected by a site × decade interaction (*F*_10,136_ = 5.263, *p* < 0.001), with densities higher at East Tektite in the 2000s and 2010s, but at Donkey Bite and West Tektite in the 1990s, and declines over time at most sites (figure 2). On a scale of metres, the densities were similar along each transect over 17 years, and the trend was similar for *Porites* spp. (figure S1 in the electronic supplementary material).

For tagged juveniles, sample sizes ranged from 1 (Yawzi Point, 2019–2020) to 104 (West Tektite, 2005–2006), with mean sample sizes (±s.e.) varying from 13.6 ± 1.7 at Donkey Bite to 53.8 ± 5.6 at West Tektite. Annual mortality varied from 19.9 ± 3.5% (Donkey Bite) to 28.1 ± 4.4% (East Tektite), but did not vary among sites (*F*_5,115_ = 1.813, *p* = 0.116).

## 4. Discussion

Recruitment hotspots have long attracted research attention because they can serve as loci of population growth and drivers of spatial variation in community structure [10,16]. As ecosystems degrade under the effects of anthropogenic disturbance, populations of many species are declining in size, creating interest in locations where they still recruit to the affected habitat [34]. As populations approach local extirpation, recruitment hotspots indirectly reveal the presence of source populations producing the propagules supporting recruitment, and directly reveal locations that might catalyze population recovery. Given the high spatio-temporal variance in recruitment of most taxa [5,35], sampling over large scales of space and time is required to detect recruitment hotspots, and suitable studies have been conducted in St. John. Reefs in this location have been studied for four decades [33,36], during which time coral cover has undergone chronic declines as well as acute losses attributed to bleaching, disease and hurricanes [36]. These events have created near-shore reefs that have stabilized at less than 4.5% coral cover and approximately 50% cover of macroalgae [33], and coral cover now is so low that even the impacts of two category five hurricanes in 2017 were not statistically detectable [37]. Against this backdrop of long-term declines in coral abundance, it is remarkable that hotspots for coral recruitment and juvenile corals have persisted over the same period. As these hotspots have not supported increasing coral cover [33,37], the recruitment bottleneck [38,39] impeding coral population recovery appears to be severe in this location.

The possibility that recruitment hotspots can be created by multiple mechanisms makes it challenging to identify the causes of any one hotspot. At a spatial scale of 100 or 1000s of metres, hotspots are likely to reflect enhanced delivery of propagules, for example by water flow [40,41]. At a scale of metres, however, they are likely to reflect biological processes such as substratum selection [8,42] or Janzen–Connell effects [43]. Given the consistent occurrence of juvenile corals along transects over 27 years (figure S1 in the electronic supplementary material), it is unlikely that recruitment hotspots in St. John are a result of substratum selection by coral larvae [44]. Instead, the expression of a recruitment hotspot on a scale of 100s of metres (i.e. Yawzi Point), suggests that hydrodynamic delivery of larvae contributes to this pattern, for example, through eddies developing from the westward flow of seawater [16,45], or the retention of locally sourced brooded larvae [46]. The flow regimes around Yawzi Point have not been measured with the rigour necessary to test this hypothesis, although the deployment of drogues within this locality [47] and the detection of high recruitment of other taxa at Yawzi Point [48] provide indirect evidence of the hydrodynamic delivery mode of hotspot formation. An implication of this mode of origin is that further settlement hotspots are likely be found along the southerly shores of other islands in the Virgin Islands where shoreline complexity juxtaposed with the prevailing flow is similar to that occurring off St. John.

In St. John, the spatial decoupling of recruitment and juvenile hotspots, with the juvenile hotspot translated eastward relative to the recruitment hotspot, highlights the complex roles of vital rates acting on different coral life stages in determining population growth. High settlement at Yawzi Point was not associated with elevated densities of juveniles, perhaps because of low recruit survivorship. At Tektite, low settlement was associated with elevated densities of juveniles, most likely because graduation from this size class was impeded by slow growth. The possibility that densities of juvenile corals were elevated at this site through enhanced survival was not supported by the similar rates of mortality at all sites (figure S2 in the electronic supplementary material). Decadal-scale reductions in growth rate of juvenile corals in this location [49] have eroded their capacity to reach adulthood, and left small corals exposed for longer periods to high mortality [50,51]. The integrated effects of mortality provide a demographic basis to the long-term reduction in density of juvenile corals (figure S2 in the electronic supplementary material).

While the detection of settlement and recruitment hotspots in low coral-cover systems (another type of coral ‘oasis’ [13]) is not a panacea for conservation designed to enhance reef restoration [52], it does show for select taxa in a region largely dominated by brooding corals that low coral cover is not caused by recruitment failure *per se* (cf. [53]). The prominence of brooding corals in St. John is important to the interpretation of the present results, because these corals release well-developed larvae that inherit their algal symbionts from their mothers, and they are capable of settling almost immediately following release [44]. These features are likely to modulate the capacity of new recruits to respond to changing conditions through their algal symbionts [54,55], and through shortened pelagic larval duration and rapid settlement, they might be more likely to be retained in eddies and settle in hotspots as described here for St. John. Since broadcasting corals show contrasting features, releasing gametes that must be fertilized and require time to develop to competency and probably take up their algal symbionts from the environment [44], they may respond in different ways to the same physical environmental condition promoting hotspots for settlement of largely brooded corals as in St. John. Caution must therefore be exerted in extrapolating the present results to other coral systems, although it is noteworthy that the octocoral *Gorgonia ventalina*, which probably reproduces by broadcast spawning [56], also recruits at Yawzi Point in unusually high numbers [48]. The detection of a recruitment hotspot at Yawzi Point rationalizes the search for causal mechanisms creating such hotspots and motivates conservation actions with the potential to change vital rates of early life stages of corals to promote population growth. Such actions might mirror the way in which coastal areas are prioritized for protection, for example, protecting beaches for sea turtles to produce hatchlings [57]. For corals, this could include exploiting hotspots as a source of coral recruits of sexual origin that could collected for out-planting in locations denuded of corals [3], or for long-term protection in a ‘Noah's arc’ [58].

## Data accessibility

The data are available at https://www.bco-dmo.org/project/835192 [59] and can be accessed through the following DOIs: 10.26008/1912/bco-dmo.854321.1, 10.26008/1912/bco-dmo.854417.1, 10.26008/1912/bco-dmo.854447.1 and 10.26008/1912/bco-dmo.854455.1.
